# Formation and Differentiation of Multiple Mesenchymal Lineages during Lung Development Is Regulated by β-catenin Signaling

**DOI:** 10.1371/journal.pone.0001516

**Published:** 2008-01-30

**Authors:** Stijn P. De Langhe, Gianni Carraro, Denise Tefft, Changgong Li, Xin Xu, Yang Chai, Parviz Minoo, Mohammad K. Hajihosseini, Jacques Drouin, Vesa Kaartinen, Savério Bellusci

**Affiliations:** 1 Developmental Biology Program, Department of Surgery, Saban Research Institute of Childrens Hospital Los Angeles, Los Angeles, California, United States of America; 2 Department of Pediatrics, Women's and Children's Hospital, University of Southern California Keck School of Medicine, Los Angeles, California, United States of America; 3 Center for Craniofacial Molecular Biology, School of Dentistry, University of Southern California, Los Angeles, California, United States of America; 4 School of Biological Sciences, University of East Anglia (UEA), Norwich, Norfolk, United Kingdom; 5 Laboratoire de Génétique Moléculaire, Institut de Recherches Cliniques de Montréal (IRCM), Montréal, Québec, Canada; Baylor College of Medicine, United States of America

## Abstract

**Background:**

The role of ß-catenin signaling in mesodermal lineage formation and differentiation has been elusive.

**Methodology:**

To define the role of ß-catenin signaling in these processes, we used a *Dermo1*(*Twist2*)*^Cre/+^* line to target a floxed *β-catenin* allele, throughout the embryonic mesenchyme. Strikingly, the *Dermo1^Cre/+^*; *β-catenin^f/−^* conditional Knock Out embryos largely phenocopy *Pitx1^−/−^/Pitx2^−/−^* double knockout embryos, suggesting that ß-catenin signaling in the mesenchyme depends mostly on the PITX family of transcription factors. We have dissected this relationship further in the developing lungs and find that mesenchymal deletion of *β-catenin* differentially affects two major mesenchymal lineages. The amplification but not differentiation of *Fgf10*-expressing parabronchial smooth muscle progenitor cells is drastically reduced. In the angioblast-endothelial lineage, however, only differentiation into mature endothelial cells is impaired.

**Conclusion:**

Taken together these findings reveal a hierarchy of gene activity involving *ß-catenin* and *PITX*, as important regulators of mesenchymal cell proliferation and differentiation.

## Introduction

During development and in adult tissues, mesenchymal cells serve as precursors to diverse cell lineages, including smooth muscle cells (SMCs), endothelial cells, pericytes, lipocytes and stromal fibroblasts. The proper generation of these cell types likely relies on the controlled amplification of lineage-restricted and non-restricted mesenchymal precursors followed by their timely differentiation into the appropriate progeny.

The developing lung provides a good system for studying the regulators of epithelial and mesenchymal cell lineage formation [1] and thus regulators of epithelial progenitor fates have been elucidated. For example, hyperactive β-catenin signaling leads to aberrant amplification of distal lung progenitor cells, partly through the regulation of *N-myc* expression [2], [3], [4], while targeted disruption of *N-myc* results in premature differentiation and reduced epithelial cell proliferation [2], [3]. ß-catenin signaling also regulates the levels of *Bmp4* and *Fgfr2b* expression in distal lung epithelium [4]. FGFR2b signaling in turn, is critical for maintenance and expansion of the pool of epithelial progenitor cells, not only in lungs, but also in developing pancreas, tooth and skin [5], [6], [7].

We and others have studied the sequential development of several lung mesenchymal lineages. The distal lung contains two distinct mesenchymal cell populations: sub-epithelial; sub-mesothelial. Sub-epithelial cells express *Ptch* and respond to epithelially-derived SHH, while transient fate analysis studies using an *Fgf10*-LacZ reporter line (here after termed *Fgf10^LacZ/+^)*, show that the sub-mesothelial cells express high levels of *Fgf10* and serve as progenitors to parabronchial smooth muscle cells (PSMCs). The PSMC progenitor status is maintained by mesothelialy-derived FGF9 [8]. With time, the PSMC progenitors relocate around the bronchi, and under the influence of an epithelially-derived signal, BMP4, differentiate into PSMCs [9]. The myogenic program is then completed along the proximal airways, where the progenitors encounter Laminin-2 and Fibronectin in the epithelial basement membrane [10], [11], [12].

Hints that ß-catenin signaling is important for the development of the mesenchyme, in addition to the epithelium, have emerged from expression pattern studies and the analysis of TOPGAL and BATGAL reporter mice. TOPGAL and BATGAL alleles serve as LEF1/TCF mediated ß-catenin signaling reporters only [13], [14] and their observed activity is restricted to the late/differentiated mesenchymal derivatives, such as the smooth muscle cells surrounding the proximal airways and in the mesenchyme around the trachea [2], [4], [11]. Furthermore, overexpression of Wnt5a has been shown to either directly or indirectly regulate *Fgf10* expression in the mesenchyme [15] while Wnt7b has been demonstrated to act on lung vascular SMCs through Frizzled 1 and LRP5 [16]. Besides LEF1/TCF mediated ß-catenin signaling, ß-catenin can also act through the PITX family of transcription factors [17], which are abundantly expressed in developing mesenchymal tissues [18]. Yet, the precise role and contribution of ß-catenin-PITX signaling axis in the early development and specification of mesenchymal lineages has not been studied in detail.

We have carried out a *Dermo1^Cre/+^*-mediated conditional inactivation (CKO) of *β-catenin* to study the role of ß-catenin signaling in mouse embryonic mesodermal lineages. In these mutants, we find multiple mesenchymally-related defects that are remarkably reminiscent of a double knock out of *Pitx1* and *Pitx2* genes [19]. By focusing on the lungs of the conditional mutant embryos and combining fate analysis and global gene expression pattern studies, we show for the first time that mesenchymal ß-catenin signaling has a dual, lineage-dependant function. It regulates the formation and amplification of *Fgf10*-expressing PSMC progenitor cells but does not affect their differentiation. Yet, it is required for proper differentiation of endothelial cells. These findings reveal a critical requirement for ß-catenin signaling in the development of multiple mesenchymal lineages.

## Results and Discussion

### Phenotypic similarities between Dermo1^Cre/+^-mediated inactivation of β-catenin and complete loss of Pitx1/2

Analysis of 327 embryos from F1 intercrosses revealed that the *Dermo1^Cre/+^; β-catenin^f/-^* conditional knockout (here on abbreviated to CKO) is lethal at embryonic day E13.5-E14.5 due to the severe cardiac (supplemental figure) and vasculogenesis-related defects. CKO embryos show a set of phenotypes that are remarkably reminiscent of *Pitx2* null embryos. These include: an arrest in turning of the body axis and defective body wall closure; partial right pulmonary isomerism; altered cardiac position with major cardiac outflow tract abnormalities classified as double outlet right ventricle (DORV) (90%) and Pulmonary truncus arteriosus (PTA) (10%) (n = 20); defective development of the mandibular and maxillary facial prominences and regression of the stomodeum (supplemental Fig. S1) [17], [18], [20], [21], [22]. Furthermore, CKO embryos exhibit minor forelimb and severe hind limb defects (supplemental Fig. S1), the latter being characteristic of the *Pitx1/2* double KO [19].

Several studies have suggested that the ß-catenin signaling pathway can induce *Pitx2* expression, and that direct binding of β-catenin to PITX2, converts PITX2 into a transcriptional activator [17]. Our in vivo findings provide the strongest genetic evidence yet for involvement of a ß-catenin-PITX axis in the formation and/or differentiation of multiple mesenchymal lineages.

Here on, we study the lung mesenchyme of CKO embryos to dissect these interactions and decipher their precise role in mesenchymal cell lineage differentiation.

### CKO of β-catenin in lung mesenchyme alters the growth and patterning of mesenchymal and epithelial cells

**Figure 1:**
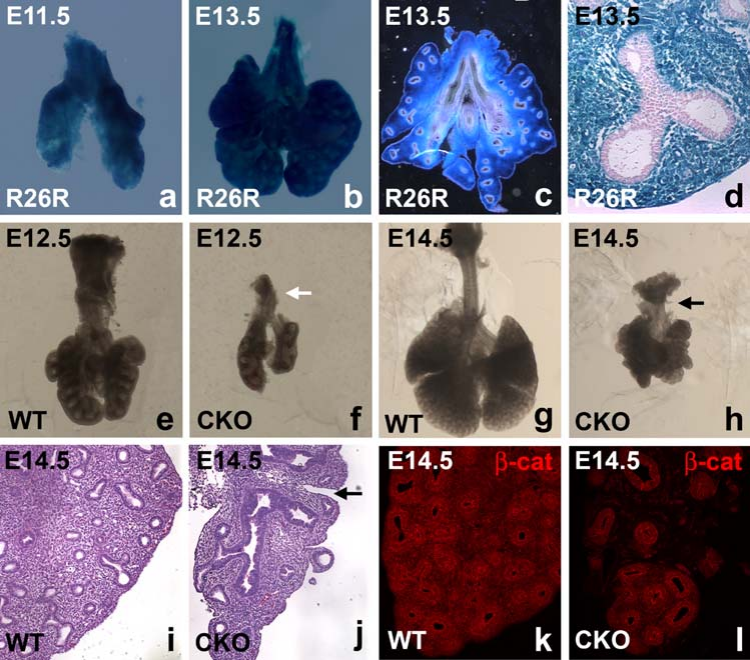
Decreased branching, partial right isomerization and epithelial proximalization in CKO lungs. (a–d) β-gal staining of *Dermo1^Cre/+^/Rosa26R* WT lungs at E11.5 and E13.5 reports Cre recombination activity in the mesenchyme of the embryonic lung. (a–b) Whole mount pictures of β-gal staining. (c) Vibratome section through E13.5 lung. (d) Paraffin section through E13.5 β-gal stained lung and counterstained with eosin. (e–h) (e) E12.5 WT and (f) CKO lungs which show decreased branching. (g) E14.5 WT lung and (h) CKO lung which shows partial right isomerization, astereotypic branching and a grape like structure, and absence of trachea. (i–j) H&E staining of sections through the left lobe of (i) E14.5 WT and (j) CKO lungs. Left lobe of CKO lung features proximalized airways and partial lobular septation. (k–l) Immunostaining for β-catenin on sections of (k) E14.5 WT and (l) CKO lungs show a mosaic deletion of β-catenin throughout the lung mesenchyme.

To monitor the onset and pattern of Cre activity in *Dermo1^Cre/+^* lungs, we crossed the *Dermo1^Cre/+^* mice [23] with *Rosa26R* reporter mice [24] and found a strong Cre-activity detectable in the mesenchyme surrounding the trachea and primary bronchi illustrated at E11.5 (Fig. 1a) and E13.5. This activity is detectable throughout the developing mesenchyme but not in the epithelium (Fig. 1b–d).

To validate the specific inactivation of *β-catenin* in the mesenchyme, we compared the pattern and levels of β-catenin expression in CKO versus wild type lungs by immunofluorescence staining. Except for occasionally a few patches, we could not detect β-catenin expression in the mesenchyme of E14.5 CKO lungs and consistent with the restriction of *Cre* expression to *Dermo1^Cre/+^* mesenchyme, epithelial β-catenin expression appeared unperturbed. Persistence of β-catenin in some small patches of lung mesenchyme is indicative of a mosaic deletion (Fig. 1l,k) and while this mosaicism may affect the severity of phenotypes and somewhat complicate our analysis, it also provides the benefit of an internal control.

Analysis at E12.5 and E14.5 showed that the *ß-catenin* CKO lungs have shortened trachea and reduced branching as well as perturbation of normal stereotypic branching patterns observed in WT lungs. Moreover, the peripheral mesenchyme was reduced. The CKO lungs also exhibit partial isomerism such that the left lobes contained inter-lobular septations – characteristic of the right side of WT lungs, but lacked an accessory lobe (Fig. 1f,h).

**Figure 2:**
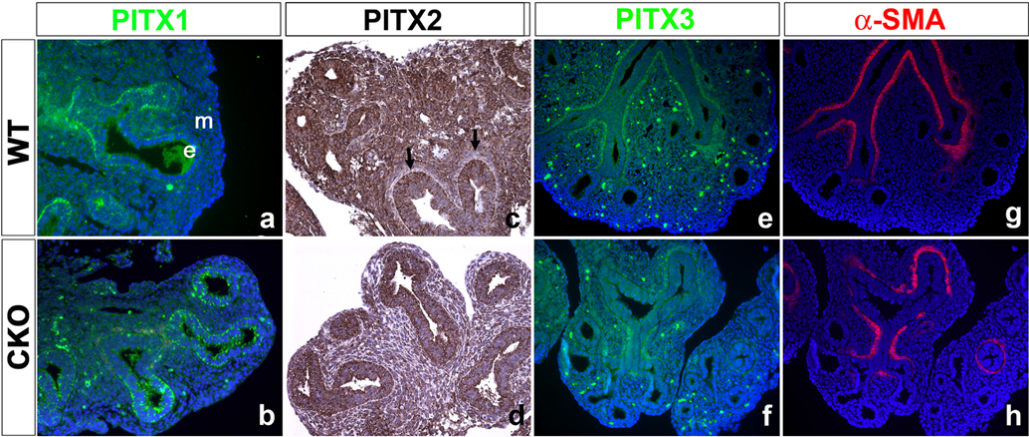
Expression pattern of PITX members in the developing wild type and mutant embryonic E13.5 lungs. (a–h) Immunostaining for PITX family members and α-SMA. (a,b) PITX1 expression in wild type lung is detected throughout the distal epithelium. (b) Expression of PITX1 in the CKO lung is not changed compared to WT. (c) PITX2 expression in wild type lung is detected throughout the epithelium as well as in the mesenchyme at the exception of the mesenchyme directly surrounding the proximal epithelium (arrows). (d) In CKO lungs, PITX2 expression is drastically reduced in the epithelium and mesenchyme. (e) In wild type lungs, PITX3 expression is present in the differentiated PSMCs adjacent to the bronchi. (f) In CKO lungs, PITX3 expression is no longer found as a continuous layer around the bronchi likely reflecting a defect in the formation of the PSMC. (g, h) SMA expression on the same section as (e) and (f).

The branching defect in CKO lungs is more severe than just the right isomerization initially described in *Pitx2* hypomorph embryos and resembles more the phenotype of lungs from mice with a complete inactivation of *Pitx2* [25]. We therefore examined the impact of CKO on PITX2 expression and asked whether its homologues, PITX1 and PITX3, are affected by loss of β-catenin. Immunostaining studies showed that PITX1 is present in both WT and CKO distal lung epithelium (Fig. 2a,b). Three *Pitx2* isoforms exist *a*, *b* and *c* the latter of which is involved in Left/right asymmetry and is only expressed on the left side of the lung [22]. PITX2 (using antibodies recognizing all three isoforms) is normally found in distal epithelium and mesenchyme but is absent from PSMC around the bronchi (arrow in Fig. 2c), and as expected, PITX2 levels were found to be drastically reduced in the CKO mesenchyme (Fig. 2d). By contrast, PITX3 is expressed exclusively in the differentiated smooth muscle cells around the bronchi in both WT and CKO lungs (Fig. 2e–h), suggestive of a switch from PITX2 to PITX3 expression upon PSMC differentiation. Although the significance of this switch needs to be elucidated, our findings indicate a major impact on PITX2 expression at the protein level.

### Loss of β-catenin signaling affects the sub-mesothelial but not sub-epithelial mesenchyme and diminishes FGF signaling

We analyzed the expression of a set of lineage and cell type-specific marker genes to examine whether all or just a subset of mesenchymal cells are affected by the loss of β-catenin signaling.

**Figure 3:**
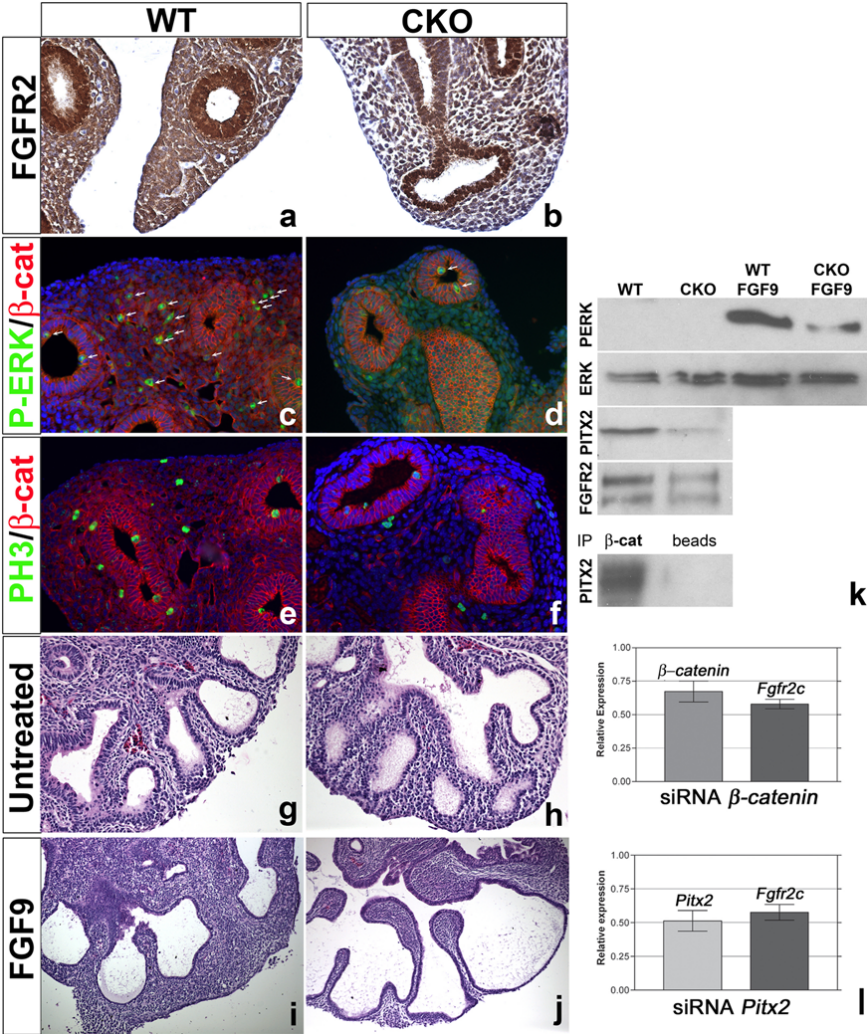
Reduced *Fgf10*, *Spry2*, *Spry4* and *Pitx2* expression in CKO lungs. Gene expression in E13.5 lungs by WMISH and LacZ staining in WT and CKO lungs (n = 3 for each probe). (a–b) *Fgf10* expression is reduced in the sub-mesothelial mesenchyme of CKO lungs. Inset: vibratome section through the distal left lobes. (c–d) *Spry2* expression is reduced in the epithelium of CKO lungs. (e–f) No difference in *Shh* or (g–h) *Ptch* expression levels in CKO lungs compared to WT lungs. Inset : vibratome section through the distal right lobes. (i–j) *Spry4* expression is reduced in the distal mesenchyme of CKO lungs illustrating reduced mesenchymal FGF9 signaling. (k–l) β-gal staining of *TOPGAL* lungs shows similar levels of TOPGAL activity in WT and CKO lungs illustrating the specificity of the *β-catenin* deletion throughout the mesenchyme. (o–n) *Pitx2* is completely ablated in CKO lungs at E13.5. (o–p) *Pitx2* expression is still present in E11.5 CKO embryos even though the *Pitx2^−/−^* phenotype is already apparent indicating that interaction of PITX2 with β-catenin is necessary for β-catenin signaling.

In the developing lungs, *Fgf10* is expressed by the PSMC progenitors, which are sub-mesothelial in origin [26]. Localized expression of *Fgf10* in the distal mesenchyme also drives the stereotypic branching observed during early lung development [27]. Except for a few patches in the right lobes, levels of *Fgf10* were greatly reduced in E13.5 CKO lungs (Fig. 3a,b) and these patches likely reflect the mosaicism of *ß-catenin* inactivation. Vibratome sections showed that in the left lobes there is almost complete absence of *Fgf10* expression (insets in Fig. 3a,b). Interestingly, we also found a marked reduction in *Spry2* expression in the distal epithelium of CKO lungs (Fig. 3c,d). As a read out, *Spry2* reduction is indicative of reduced epithelial FGF signaling and correlates with decreased epithelial branching [26].

We then examined whether the sub-epithelial distal mesenchymal domain, was similarly affected. Expression of *Shh* (Fig. 3e,f) and *Ptch* (Fig. 3g,h) was assessed but no differences between WT and CKO sub-epithelial mesenchyme were observed. However, the sub-mesothelial mesenchymal domain in CKO lungs containing the *Fgf10*-expressing progenitors was lost (Fig. 3g,h inset). Taken together, this suggests that β-catenin signaling specifically regulates the *Fgf10*-expressing progenitor cells in the sub-mesothelial mesenchyme.

We have previously shown that mesenchymal FGF signaling is important for the expression of *Fgf10* within, and for the ensuing survival and proliferation of distal PSMC mesenchymal progenitors [28]. Mesenchymal FGF signaling induced by FGF9 is also important for preventing the differentiation of PSMC progenitors [8], [28]. *Spry4*, expressed in the distal mesenchyme is a faithful reflector of levels of FGF signaling in this tissue compartment [28]. Interestingly, we found that levels of *Spry4* were also reduced in CKO lungs suggestive of reduced levels of mesenchymal FGF signaling (Fig. 3i,j).

We then compared the expression of *Bmp4* in the epithelium of WT and CKO lungs as BMP4 engages the *Fgf10* expressing progenitors into the smooth muscle cell lineage [9], [29]. However, no difference in *Bmp4* expression between WT and CKO lungs was observed (data not shown) suggesting that the differentiation of the PSMC progenitors could occur normally in CKO lungs.

### Mesenchymal defects in CKO lungs do involve a gradual loss of PITX

Next, we used an imported TOPGAL reporter allele to examine the level and distribution of LEF1/TCF-mediated β-catenin signaling. At E13.5, TOPGAL activity was restricted to epithelium, in both WT and CKO lungs, with no discernable difference in levels (Fig. 3k,l). This finding further confirms that in the CKO mice, *β-catenin* is specifically deleted in the mesenchyme but importantly, it also demonstrates that mesenchymal β-catenin signaling at E13.5 does not rely primarily on LEF1/TCF transcription factors.

As the ß-catenin-PITX2 pathway has previously been shown to regulate *Pitx2* expression itself, both at the level of transcription [17] and mRNA stability [30], we compared the levels of *Pitx2* expression in CKO and WT lungs by in situ hybridization. Using a riboprobe that detects all three isoforms of *Pitx2*, we found that at E13.5, CKO lungs show an absence of *Pitx2* expression (Fig. 3m,n) further validating the IHC-derived observations (Fig. 2a,b). Previous studies reported that in the embryonic lung both PITX2 and LEF1 are present in the mesenchyme suggesting potential redundant function between these 2 transcription factors [31], [32], [33]. However, contrarily to *Pitx2* inactivation which leads to abnormal lung development, *Lef1* inactivation does not affect lung development [34], indicating that PITX2 can compensate for the loss of LEF1 but not vice versa. This observation argues for a privileged ßcatenin-PITX2 signaling axis in the embryonic lung mesenchyme.

Remarkably, at E11.5, CKO embryos already exhibit a *Pitx1/2* KO-like phenotype even though *Pitx2* expression is not fully extinguished at this stage (Fig. 3o,p). This observation indicates that loss of mesenchymal β-catenin per se does not immediately result in loss of *Pitx2* expression, but a β-catenin/PITX2 interaction is required for mesenchymal ß-catenin signaling.

### Fgfr2 is a downstream target of β-catenin signaling in the mesenchyme

**Figure 4:**
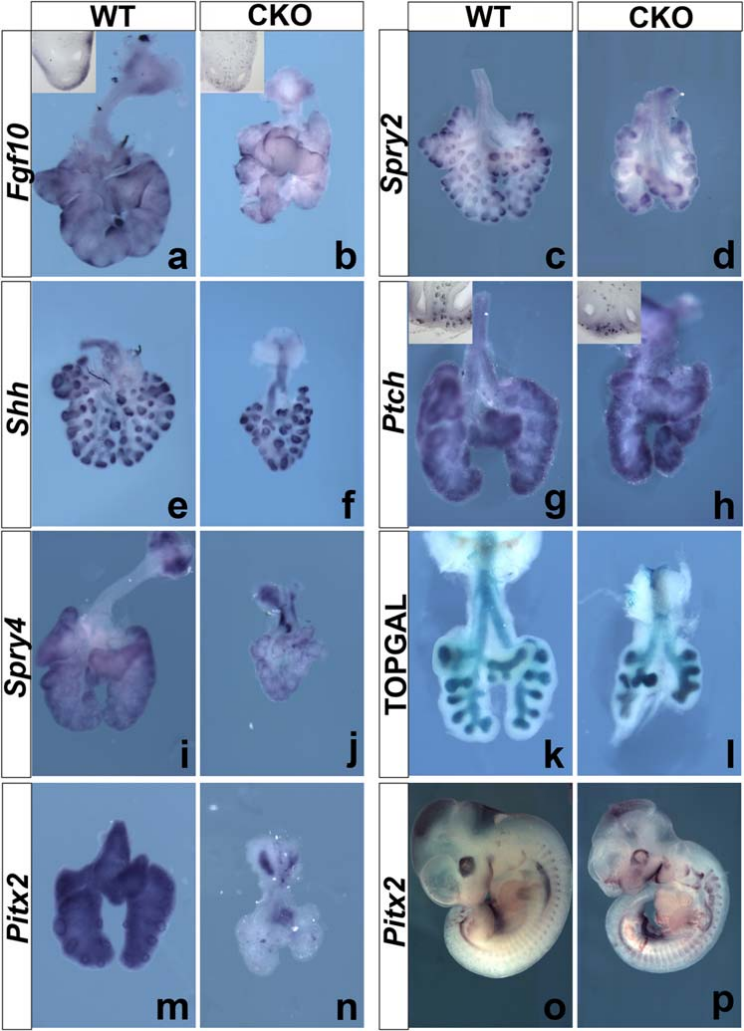
Reduced FGFR expression, P-ERK and proliferation in CKO mesenchyme. (a–b) Immunohistochemistry. Reduced expression for FGFR2 in E13.5 CKO lung mesenchyme. Expression in the CKO epithelium is unaffected. (c–d) Immunofluorescence for phospho-ERK (P-ERK) in green (arrows) and β-catenin in red, DAPI (Blue). (e–f) Immunofluorescence for phospho-HistonH3 (PH3) in green and β-catenin in red, DAPI (Blue) (g–j) H&E stained sections through E12.5 WT and CKO lungs cultured for 48h in vitro in the presence or absence of 200 ng/ml FGF9. (g,i) WT lungs grown in the presence of FGF9 (i) show decreased branching, dilation of the epithelium and overproliferation of the distal mesenchyme compared to untreated lungs (g). (h,j) CKO lungs grown in the presence of FGF9 (j) only show an epithelial effect and dilation of the epithelium while proliferation of the distal mesenchyme remains absent. (k) Upper part: western blot analysis on primary culture of WT and CKO lung mesenchyme treated or not with FGF9 with P-ERK, total-ERK, PITX2 and FGFR2 antibodies. Lower part: Co-Immunoprecipitation of PITX2 with β-catenin from primary culture of wild type lung mesenchyme cultured in the presence of 10 mM LiCl. Absence of co-immunoprecipitation of PITX3 with β-catenin from primary culture of wild type lung mesenchyme. (l) Relative *β-catenin*, *Fgfr2* and *Pitx2* expression levels in primary cultures of mesenchyme treated with siRNA to *β-catenin* (top) and *Pitx2* (bottom) analyzed by real time PCR.

The observed reduction in *Fgf10* and *Spry4* expression, indicative of reduced FGFR2C signaling in CKO lung mesenchyme, led us to investigate its mechanism. Shu et al., (2005) reported that inactivation of *β-catenin* in the distal lung epithelium, leads to a down-regulation of *Fgfr2* receptor in the epithelium. By inactivating *β-catenin* in the lung mesenchyme, we might expect to observe a similar down-regulation of *Fgfr2* in the mesenchyme. Using immunohistochemistry, we found that indeed FGFR2 expression is reduced in the mesenchyme but not epithelium of CKO lungs (Fig. 4a,b). Interestingly, FGFR2 expression is also reduced in *Pitx2^−/−^* lungs (supplemental Fig. S2). In this case, however, FGFR2 expression was also reduced in the epithelium, as *Pitx2* deletion was not mesenchyme specific. To further address how ß-catenin signaling in the mesenchyme regulates *Fgfr2* expression, we silenced *β-catenin* and *Pitx2* expression using siRNA in primary cultures of WT mesenchyme and monitored using real time PCR the levels of *Fgfr2* expression. Using siRNA in primary cultures of lung mesenchyme, a downregulation in *β-catenin* expression of 33%±8 (n = 3, *P = *0.03) compared to scrambled led to a corresponding downregulation in *Fgfr2* expression of 40%±4 (n = 3, *P = *0.003) (Fig 4l). Similarly, a downregulation in *Pitx2* expression of 50%±8 (n = 3, *P = *0.01) compared to scrambled led to a corresponding downregulation in *Fgfr2* expression of 40%±6 (n = 3, *P = *0.009) (Fig. 4l).

Next, we set out to investigate if this relationship is reflected in functional assays. We quantified the distribution of P-ERK positive cells and found much fewer P-ERK positive cells in the mesenchyme of CKO lungs wherever *β-catenin* was deleted (0.1±0.1% vs. 1±0.2%, n = 3, *P* = 0.03) (Fig. 4d,c arrows). The number of P-ERK positive cells in the epithelium was also reduced in CKO lungs and this correlates with the reduction in *Spry2* expression (1±0.3% vs. 3±0.35%, n = 3, *P = *0.03). There was also a clear reduction in the number of mitotic cells, as determined by Phospho-Histone H3 (PH3) staining, in mesenchyme (0.2±0.1% vs. 1.1±0.1%, n = 3, *P* = 0.02) (Fig. 4f,e) and epithelium (2±0.2% CKO vs. 3.2±0.2% WT, n = 3, *P* = 0.03) of CKO, when compared to WT.

Interestingly, no significant differences in P-ERK or PH3 could be found in areas of CKO lung mesenchyme that lacked recombination (P-ERK 1.3±0.1% vs. 1.1±0.2% *P* = 0.2) (PH3 1.5±0.2% vs. 1.1±0.1%, n = 3, *P* = 0.2), demonstrating that the effect of *β-catenin* deletion in CKO lungs on mesenchymal proliferation and ERK phosphorylation is cell autonomous.

We also tested the response of CKO and WT lung explants to FGF9 treatment, which in a normal scenario would stimulate the proliferation of sub-mesothelial mesenchyme and cause dilation of the epithelium, effects that are brought about by FGFR2 signaling [8], [35] (Fig. 4i,g). Treatment of CKO lungs lead to dilation of the distal epithelium (Fig. 4j,h) but the corresponding mesenchyme was markedly thinner, when compared to the FGF9-treated WT lungs (Fig. 4j,i), indicative of a reduced mesenchymal response to FGF9 involving reduced FGFR2 expression. In support of this, we found much lower level of ERK phosphorylation in CKO explants treated with FGF9, and reduced expression of FGFR2 itself in non-treated cultures, when compared to WT (Fig. 4k).

In this system, we also found that PITX2 expression is decreased in CKO lung mesenchyme. Finally, a set of immunoprecipitation studies indicated that PITX2 is not only a downstream target of β-catenin signaling in the lung but also binds to β-catenin (Fig. 4k). However, under similar experimental conditions, we could not find an interaction between β-catenin and PITX3 proteins (Fig. 4k).

Taken together these results support the notion that *Fgfr2* is a specific downstream target gene in the β-catenin/PITX2 pathway of the mesenchyme. Conditional deletion of *β-catenin* in the lung mesenchyme results in the functional inactivation of the FGFR2c signaling pathway. In turn, this affects the sub-mesothelial mesenchymal domain containing the PSMC progenitor cells, which is known to depend on mesenchymal FGF signaling for its maintenance and proliferation [8], [28].

### Reduction of PSCM cells correlates with loss of c-Myc expression

**Figure 5:**
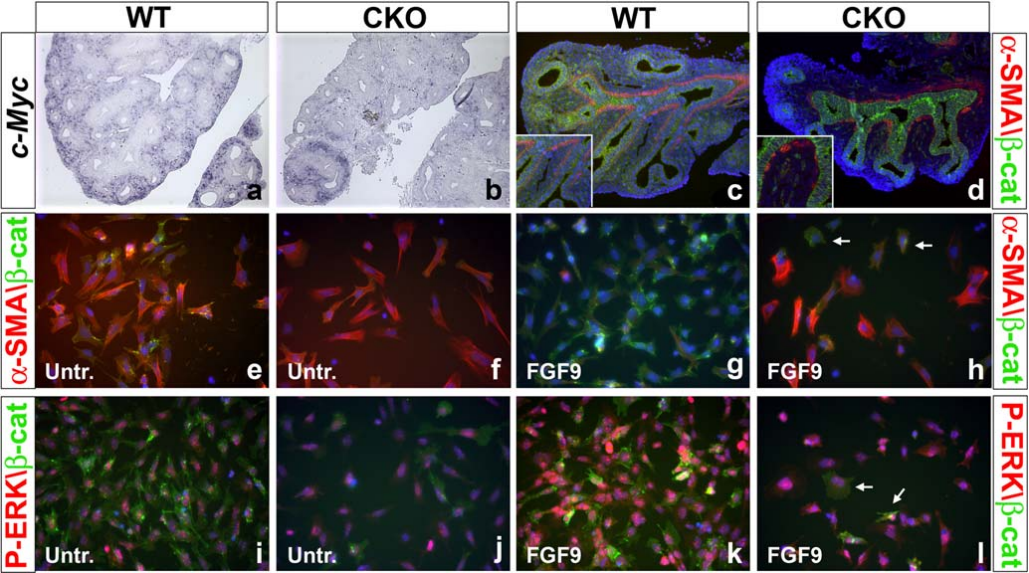
Reduced *c-Myc* expression and lack of PSMC progenitor amplification in CKO lungs. (a-b) Section RISH for *c-Myc* on E14.5 WT and CKO lungs. Expression of *c-Myc* is lost in CKO lung mesenchyme were *β-catenin* is completely deleted but remains the same were β-catenin expression is unaltered. These sections are adjacent to the ones represented in Fig. 1k,l illustrating mosaic β-catenin deletion. (c-d) Immunofluorescence for β-catenin (green) and α-SMA (red) on sections through E13.5 WT and CKO lungs. Absence of β-catenin in CKO lung mesenchyme and patchy α-SMA expression around the bronchi (d) and high magnification inset in (d). (e–f) Untreated primary cultures of mesenchyme from both WT and CKO lungs spontaneously differentiate into smooth muscle cells in vitro. (g–h) Primary cultures from WT lungs treated with FGF9 fail to differentiate into smooth muscle cells (g) while primary cultures from CKO lungs (h) are not affected in their differentiation after FGF9 treatment. (i–l) Immunofluorescence for β-catenin (green) and P-ERK (red) on primary culture of WT and CKO lung mesenchyme treated or not with FGF9. Upon FGF9 treatment, Note the drastic increase in P-ERK expression in WT cells in comparison to CKO cells.

The loss of proliferation noted in CKO lungs led us to measure the levels of *c-Myc* expression, which is known to be a ß-catenin/PITX2 signaling target gene [17] and key regulator of cell proliferation [36]. As shown in Fig. 5a, *c* *-Myc* is normally expressed in the mesenchyme but its expression levels are drastically reduced both in CKO lungs (Fig. 5a,b) and in *Pitx2^−/−^* lungs (supplemental Fig. S2). *c-Myc* expression was maintained in the regions where *β-catenin* was not deleted (Fig. 1l), serving therefore as an internal control and indicating further that these effects are cell autonomous. Together with the proliferation data presented earlier (Fig. 4c,d), these results support the conclusion that ß-catenin signaling drives the proliferation of *Fgf10*-expressing sub-mesothelial cells.

Next, we examined the consequence of this reduced cell proliferation on parabronchial smooth muscle formation around the bronchi, since sub-mesothelial mesenchymal progenitors contribute to these muscles. Immunofluorescent staining with SMA-specific antibodies revealed that the continuity of the PSMC layer around the bronchi of the CKO lungs is compromised (Fig. 5d,c). The presence of some PSMC cells indicates that loss of *β-catenin* per se does not necessarily affect the differentiation of the progenitors into smooth muscle cells, but reflects a reduction in the pool of PSMC progenitors. Moreover, the presence of PSMCs indicates that some expansion of the PSMC progenitor pool did occur.

We also tested the potential of CKO mesenchymal cells to differentiate into smooth muscle cells in vitro and found this unperturbed [37] because, like WT cells, cultured CKO cells readily differentiated into SMC (Fig. 5e,f). Raising the level of FGF signaling in the mesenchyme by FGF9-treatment normally inhibits the SMC differentiation [11], but FGF9 stimulation had no such effect on CKO-derived cells (Fig. 5g,h). We also carried out a similar IF experiment to detect P-ERK expression. Treatment of WT cells with FGF9 results in a 116 %±8.7 (n = 3, *P = *0.005) increase in P-ERK (Fig. 5k,i). By contrast, treatment of CKO cells with FGF9 leads only to a modest 70%±3.5 (n = 3, *P = *0.001) increase in P-ERK levels (Fig. 5l,j). These observations demonstrate further that CKO cells have the capacity to differentiate into smooth muscle cells, but are unable to sufficiently respond to FGF9's inhibitory effect, most likely because FGFR2 is functionally downregulated following conditional deletion of *β-catenin* (Fig. 4a,b,k).

### Mesenchymal β-catenin signaling is essential for the amplification of the Fgf10 expressing PSMC progenitors and differentiation of the angioblasts into mature endothelial cells

**Figure 6:**
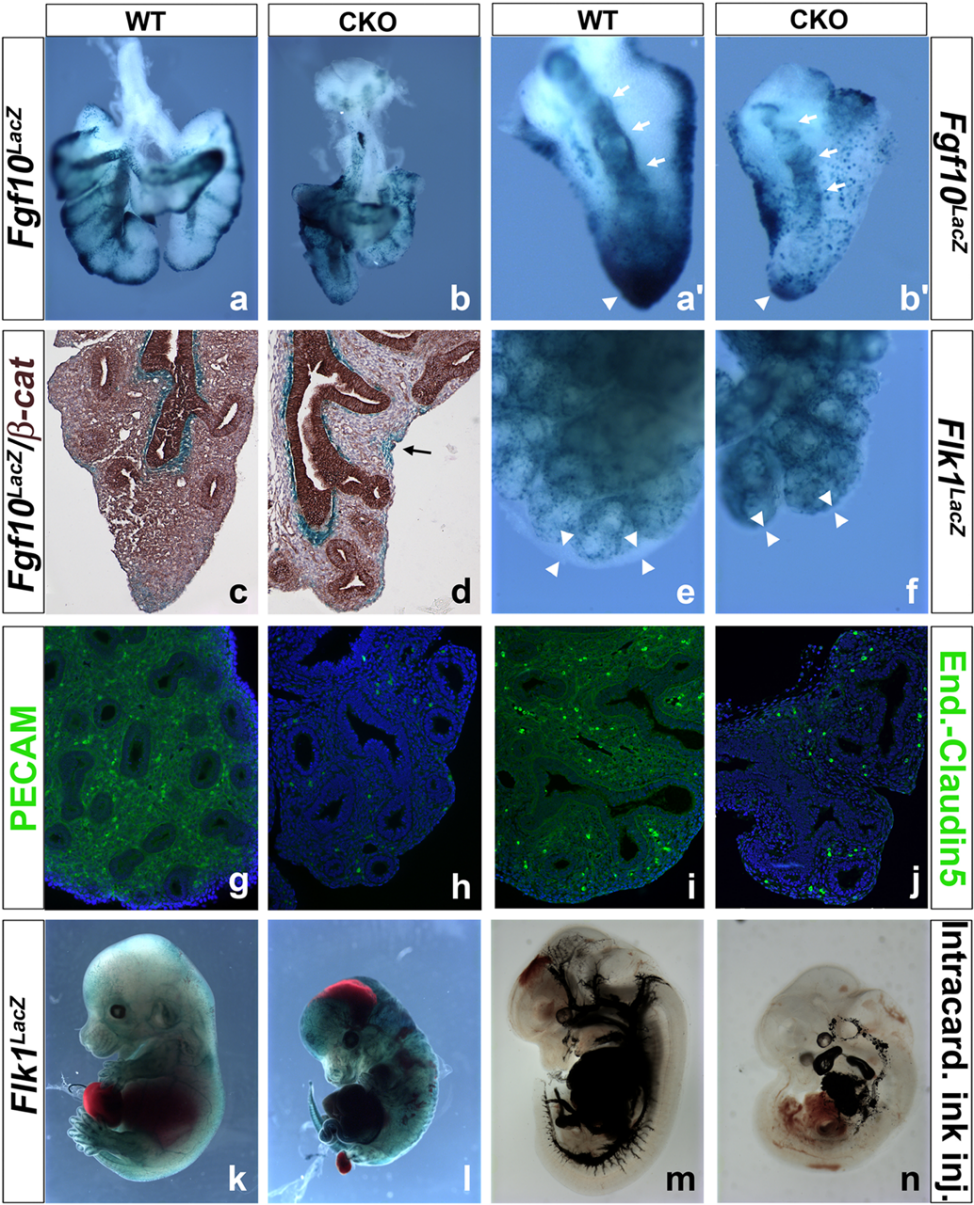
Lack of PSMC progenitor amplification and failure of endothelial progenitor cell differentiation. (a–b') β-gal staining on WT and CKO lungs crossed with the *Fgf10^LacZ^* reporter line. β-gal staining in the CKO lung (b) is severely reduced and the presence of single progenitor cells are apparent. (a'–b') Close up on the accessory lobe shows the presence of single *Fgf10^LacZ^* positive PSMC progenitor cells in the distal mesenchyme of the CKO lung. Lineage tracing of the *Fgf10^LacZ^* positive PSMC progenitor cells in CKO lungs (b') shows fewer cells are relocating around the bronchi compared to the WT lungs (a'). (c–d) IHC for β-catenin (brown staining) on paraffin sections of WT and CKO lungs crossed with the *Fgf10^LacZ^* reporter. (d) Presence of β-gal staining in the distal mesenchyme in the absence of β-catenin expression (arrow). (e–f) β-gal staining on WT and CKO lungs crossed with the *Flk1^LacZ^* reporter line. High magnification of E13.5 left lobes show an increase in *Flk1^LacZ^* expression in the CKO lung compared to WT lungs. Arrowheads illustrate the reduction in size of the sub-mesothelial mesenchymal domain containing the *Fgf10* expressing PSCM progenitors and in which no Flk1-positive cells are present. (g–h) Immunofluorescence staining for PECAM on E14.5 WT and CKO lungs. Absence of PECAM in CKO lungs (f). (i–j) Immunofluorescence staining for endothelial-Claudin5 on E14.5 WT and CKO lungs. Absence of endothelial-Claudin5 in CKO lungs (f). (k–l) β-gal staining on E13.5 WT and CKO embryos crossed with the *Flk1^LacZ^* reporter line. CKO embryos (l) show and increased expansion of *Flk1^LacZ^* positive angioblasts throughout the embryonic mesenchyme compared to WT embryos (k). (m–n) Intracardiac India ink injection of E13.5 WT and CKO embryos. CKO embryos show defects in vasculogenesis and leakage of India ink from premature blood vessels is apparent (n) compared to WT embryos (m).

So far, we have provided experimental evidence suggesting that CKO deletion of *β-catenin* in the lung mesenchyme perturbs the amplification but not the differentiation of the PSMC progenitors into smooth muscle cells. To directly visualize the fate of the *Fgf10*-expressing progenitors, we crossed our mutant mice with a previously published *Fgf10^LacZ^* enhancer-trap line [38]. Due to the stability of the LacZ protein, this line can be used to lineage trace transiently the *Fgf10* expressing PSMC progenitors [9]. CKO lungs showed a marked reduction in *Fgf10/*LacZ expressing progenitors in the distal mesenchyme at E13.5 vs. WT lungs (Fig. 6b,a). Close analysis of the accessory lobe further illustrated the presence of single progenitor cells in the distal mesenchyme of the CKO lung (Fig 6b',a' arrowhead) and patchy LacZ expression around the bronchi compared to WT lungs (Fig. 6b',c' arrows). Immunhistochemistry for β-catenin on paraffin sections of CKO lungs crossed with the *Fgf10^LacZ^* reporter reveals that expression of *Fgf10* in the distal mesenchyme of CKO lungs is not due to the lack of recombination of the *ß-catenin^flox^* allele (Fig. 6c,d). This confirms our previous observation that ß-catenin signaling in the lung mesenchyme is important for the amplification of the PSMC progenitors or transient amplifying cells. We then examined whether the loss of ß-catenin signaling affects the differentiation of the lung mesenchyme into endothelial cells. For this, CKO lungs were generated in a *Flk1^LacZ^* reporter background [39], which expresses *LacZ* under the control of the endogenous *Flk1* promoter. Flk1 is an early marker of angioblast and its expression is maintained in mature endothelial cells [39]. Interestingly, *Flk1* expression was highly upregulated throughout the entire CKO embryo (Fig. 6k,l) including the lungs (Fig. 6e,f). Ablation of mesenchymal ß-catenin signaling therefore did not seem to interfere with the specification and amplification of the angioblast. However, PECAM and endothelial-Claudin5 staining on CKO lungs vs. WT lungs revealed an impaired differentiation of the angioblasts into mature endothelial cells and blood vessels in the CKO lungs vs. WT lungs (Fig. 6g–j). We also examined the pattern of vasculature by injecting India ink in the left ventricle of the CKO and WT hearts, only to find a clear defect in vasculogenesis throughout the CKO embryo (Fig. 6m,n). Leakage of India ink from abnormal and immature blood vessels could be observed throughout the CKO embryo. A similar underdeveloped vascular system was also observed in *Pitx2^−/−^* embryos after Intracardiac India ink injection (supplemental Fig. S2). However, PECAM staining could still be detected in endothelial cells of *Pitx2^−/−^* embryos (data not shown) indicating a possible redundancy with other PITX or LEF1/TCF transcription factors. Our results indicate that inactivation of *β-catenin* in the mesenchyme inhibits the differentiation of angioblasts into mature endothelial cells. However, it is important to note that ablation of *β-catenin* in mature endothelial cells using the *Tie2^Cre^* driver line did not affect vasculogenesis and angiogenesis, or PECAM expression [40]. *Tie2* expression starts later during endothelial cell differentiation and so our data suggests that ß-catenin signaling is an important regulator of early endothelial cell development [41], [42].

### Mesenchymal β-catenin signaling controls the amplification of PSMC progenitors in the sub-mesothelial mesenchyme

**Figure 7:**
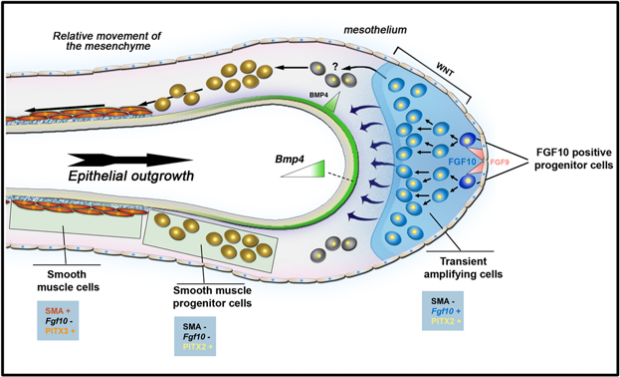
Model for lineage differentiation of the PSMCs. *Fgf10* and *Pitx2* expressing PSMC progenitors are located in the submesothelial mesenchyme and respond to FGF9 and β-catenin signaling to amplify and remain undifferentiated [8], [9], [28], [35]. As the epithelium grows out, the PSMC progenitors come in contact with BMP4 secreted by the epithelium. These cells then stop expressing *Fgf10* and get committed to the PSCM lineage [9]. When these cells eventually spread out on the epithelial basement membrane containing Fibronectin [11], they differentiate into mature PSMC and start to express α-SMA and switch from PITX2 to PITX3 expression.

Figure 7 summarizes and places our findings in the context of mesenchymal cell differentiation in the lung. Previously, we have shown that *Fgf10*-positive cells (represented in blue in model Fig. 7c) present in the sub-mesothelial mesenchyme can serve as PSMC progenitors [9], and that their proliferation is driven by FGF9 produced by the mesothelium [8], [28]. Here, we demonstrate that the amplification of these transient amplifying cells relies on β-catenin signaling via PITX2. Moreover, this important role of β-catenin signaling involves the regulation of FGFR2c and *c-Myc* expression in that loss of *β-catenin* diminishes the response of sub-mesothelial mesenchyme to mesothelially-derived FGF9, resulting in a dramatic reduction in the pool of *Fgf10*-expressing PSMC progenitors, contained within the sub-mesothelial mesenchyme. This impairment has also been observed in the lungs of *Fgf9^−/−^* embryos [35], [43]. Interestingly, *β-catenin* abrogation in the mesenchyme specifically interferes with the amplification of the PSMC progenitors but does not affect, at least in vitro, their differentiation into smooth muscle cells.

As PSMC progenitors relocate around the bronchi, they are exposed to high levels of epithelial BMP4, and eventually stretch out on the epithelial basement membrane which engages them into the smooth muscle cell lineage [9], [11], and there appears to be a switch from PITX2 to PITX3 expression.

Our data suggest that PITX2 and 3 have distinct effects on cell fate and seem to be differently affected by the deletion of *β-catenin*. We propose that mesenchymal β-catenin signaling in the sub-mesothelial mesenchyme acting at least in part via PITX2 is necessary for the amplification of the PSMC progenitors or at least the proliferation of the transient amplifying cells derived from the *Fgf10* expressing PSMC progenitors. Use of the *Fgf10^LacZ^* reporter shows that single *Fgf10*/LacZ positive PSMC progenitors are present in the distal mesenchyme of CKO lungs. The presence of these cells may indicate that β-catenin signaling is required for asymmetrical division of these progenitors into PSMC precursor cells. Deletion of *β-catenin* in the progenitor cells leads to impaired formation of transient amplifying cells, revealing the single *Fgf10*/LacZ expressing PSMC progenitors in the CKO lungs. However, deletion of *β-catenin* in the progenitors or the transient amplifying cells does not inhibit their differentiation into smooth muscle cells, which coincides with a switch in expression from PITX2 to PITX3. On the contrary, FGF9 is no longer capable of maintaining the undifferentiated state of mesenchymal cells, effectively allowing them to differentiate prematurely.

Paradoxically, ß-catenin signaling seems to play the opposite role in the other major mesodermally derived cell lineage, the endothelial cell lineage. In the whole CKO embryo and in the lung in particular, we observe an amplification of *Flk1*-positive angioblasts. We propose that absence of *β-catenin* prevents them from differentiating into mature endothelial cells. Interestingly *c-Myc*, which is down-regulated in the CKO lung mesenchyme, has also previously been shown to be essential for vasculogenesis and angiogenesis during development and tumor progression [44].

In conclusion, our data indicate that ß-catenin signaling in the undifferentiated lung mesenchyme is mediated by members of the PITX family of transcription factors and exhibits a duality in effect, being necessary for the amplification of the *Fgf10*-expressing PSMC progenitors on the one hand and the proper differentiation of angioblasts into mature endothelial cells on the other.

## Materials and Methods

### Transgenic embryos

*β-catenin^f/f^*; *CMV-Cre*, *Rosa26R*, *Flk1^LacZ/+^* and TOPGAL, mice were obtained from The Jackson Laboratory. *Dermo1^Cre/+^* mice were a kind gift from Dr. David Ornitz [23], *Fgf10^LacZ/+^* mice were a generous gift from Dr. Robert Kelly [38] and *Pitx2^−/−^* embryos were a kind gift of Dr. James Martin [21]. *β-catenin* heterozygotes were obtained by crossing floxed *β-catenin* mice with CMV-Cre mice. *β-catenin^+/−^* mice were crossed with *Dermo1^Cre/+^* mice to obtain double heterozygous males, which were then crossed with *β-catenin* ^f/f^ females. Also *β-catenin^f/f^/Rosa26R^+/+^; β-catenin^f/f^/TOPGAL^+/+^; β-catenin^f/f^/Flk1^Lacz/+^* and *β-catenin/Fgf10^LacZ/+^* mice were created by intercrossing *β-catenin^f/f^* mice with the respective mouse reporter lines.

### β-galactosidase staining

Tissues containing *Rosa26R*, *Flk1^LacZ^*, *TOPGAL* or *Fgf10^LacZ^* alleles were dissected and β-galactosidase staining was performed as previously described [11].

### In situ hybridization

WMISH was performed like previously described [28]. Paraffin sections of embryonic lungs were hybridized using a protocol adapted from [45]. The following mouse cDNAs were used as templates for the synthesis of digoxigenin-labeled riboprobes: a 1.5 kb full-length mouse *Bmp-4*, a 642 bp *Shh*, a 584 bp fragment of *Fgf10*, a 1.1 kb *Spry4* probe, a 948 bp full-length mouse *Spry2* cDNA, a 841 bp fragment of *Ptch*, a 559 bp fragment from *Pitx2* present in all 3 *Pitx2* isoforms (cloned by RT-PCR using primers *Pitx2*-F gcagaggactcatttcacta and *Pitx2-R* tataaacgtacggaggagtc) and a 201 fragment of *c-Myc* (cloned by RT-PCR using primers *c-Myc-F* accaacaggaactatgacctc and *c-Myc-R* aaggacgtagcgaccgcaac).

### Isolation of mesenchymal cells

Mesenchymal cells from E13.5 WT and CKO lungs were isolated according to [46].

### Immunochemistry

E13.5 CKO and WT lung mesenchymal cells were grown on 8 well permanox Lab-Tek chamber slides in the presence of FGF9 (200 ng/ml) for 24 hours. The cells were fixed for 30 minutes with 4% paraformaldehyde and subsequently washed with PBS. Lungs were fixed in 4% PFA washed in PBS, dehydrated and paraffin embedded. Lung sections and slides were treated with a monoclonal anti α-smooth muscle actin antibody (Ab), clone 1A4, Cy3 conjugated (from Sigma®) at 1∶200, anti-β-catenin Ab (BD biosciences) 1∶200, anti-PH3 Ab (cell signaling) 1∶100, anti-PERK Ab (cell signaling) 1∶100, anti PECAM Ab (BD biosciences) 1∶50, anti FGFR2 (Bek) Ab (Santa Cruz) 1∶50, anti-Nkx2.1 Ab (TTF1) (Neomarkers), PITX1 and PITX3 were generated in Dr. Drouin's laboratory and were described previously [47], [48]. Ab against PITX2 were a kind gift of Dr. Hjalt [33]. Alternatively, PITX3 Ab from (Zymed) were also used. Dako cytomation CSAII signal amplification system was used for FGFR2 immunohistochemistry and slides were mounted using DPX. For immunofluorescence, FITC and CY3 conjugated F(ab')_2_ fragments were purchased from Jackson Immunoresearch and slides were mounted with DAPI containing Vectashield®.

### Western blot and immunoprecipitation

Western blot and Immunoprecipitation studies using antibodies against P-ERK, total ERK, FGFR2 and PITX2 were carried out as previously described [9]. Cell lysates from primary culture of WT or CKO lung mesenchymal cells were immunoprecipitated with β-catenin antibodies and analyzed by western blot for the presence of a PITX2/β-catenin or PITX3/β-catenin complexes. Primary cultures were grown in the presence of 10 mM LiCl to study the interaction of ß-catenin and PITX2 or were serum starved to study the interaction of ß-catenin with PITX3.

### siRNA transfection

Pre-validated siRNA pools targeting mouse *β-catenin* and *Pitx2* Dharmacon (ON-TARGETplus SMARTpool) were used. *β-catenin* and *Pitx2* siRNA or scrambled siRNA were transfected into primary cultures of lung mesenchymal cells using Lipofectamine LTX (Invitrogen) according to the manufacturer's instructions. Mesenchymal cells at a density of 1×10^4^ cells per well in 12-well plates were transfected in triplicate with 50 µM siRNA. The percentage of silencing of *β-catenin* and *Pitx2* and the effect on *Fgfr2c* expression were detected by real-time PCR as previous described [28].

### Proliferation and PERK study

Proliferation and PERK assays were performed as previously described in [28]. Quantification of PERK levels in Fig. 5 in primary cultures of mesenchyme was performed using ImageJ software (NIH).

### Organ culture

Lung explants isolated from E12.5 WT and CKO embryos were cultured and treated with 200 ng/ml FGF9 (R&D systems) as previously described [8].

### Intracardiac ink injections

India ink was injected intracardially with custom made glass pipettes (12 µm opening) at E13.5. After injections, embryos were fixed in 4% formaldehyde for 12 hours, dehydrated and cleared in benzyl benzoate:benzyl alcohol (2∶1).

## Supporting Information

Figure S1 Inactivation of *β-catenin* in the mesenchyme resembles the phenotype of *Pitx2* null embryos. (a) Frontal images of control and CKO embryos at E13.5. (b-c) Ectopic hearts and visceral organs in CKO's vs. WT with leftward displacement of ventricles (pseudo-colored in green). (d,e) β-galactosidase staining of WT or CKO embryos containing the Fgf10LacZ allele. CKO embryos display severe hind limb defects with no detectable LacZ/Fgf10 expression (inset in e). (f,g) Defective development of the mandibular and maxillary facial prominences and regression of the stomodeum. (h, i) Altered cardiac position with major cardiac outflow tract abnormalities in CKO heart (i) compared to WT (h). In WT, the pulmonary trunk (PT) rises from the right ventricle (rv) and is separated from the aorta (Ao), which rises from the left ventricle, by the aortic-pulmonary septum (rv and PT pseudo-colored in blue, lv and Ao in yellow). (j-r) Histology of control (j-l) and mutant (m-r) at E13.5 (transverse sections on comparable axial levels from rostral to caudal). Most mutants display double outlet right ventricle or DORV (m, n), i.e., both the aorta and pulmonary trunk originate from the right ventricle. A subset of mutants demonstrates a single outflow tract rising from the right ventricle, i.e., they display Pulmonary truncus arteriosus or PTA (p-r). Leftward orientation of the heart is evident in all mutants; moreover the right ventricle is largely located above the left ventricle (m-r).
(4.86 MB TIF)

Figure S2 Comparative analysis of the *Pitx2^−/−^* phenotype. (a-b) Images of control and *Pitx2^−/−^* embryos at E12.5. (c-d) Immunohistochemistry. Reduced expression for FGFR2 in E12.5 *Pitx2^−/−^* lung mesenchyme and epithelium (d) compared to WT lungs (c). (e-f) Section RISH for *c-Myc* on E12.5 WT and *Pitx2^−/−^* lungs. Expression of *c-Myc* is reduced in *Pitx2^−/−^* lung mesenchyme (f) compared to WT lung mesenchyme (e). (g-h) Intracardiac India ink injection of E12.5 WT and *Pitx2^−/−^* embryos. *Pitx2^−/−^* embryos show defects in vasculogenesis and leakage of India ink from premature blood vessels is apparent (g) compared to WT embryos (h).
(10.05 MB TIF)
