# Supplementary figures and images for: Formation and Differentiation of Multiple Mesenchymal Lineages during Lung Development Is Regulated by β-catenin Signaling
Source: PLoS One. 2008 Jan 30;3(1):e1516. doi: 10.1371/journal.pone.0001516 (PMC2211394; doi:10.1371/journal.pone.0001516)

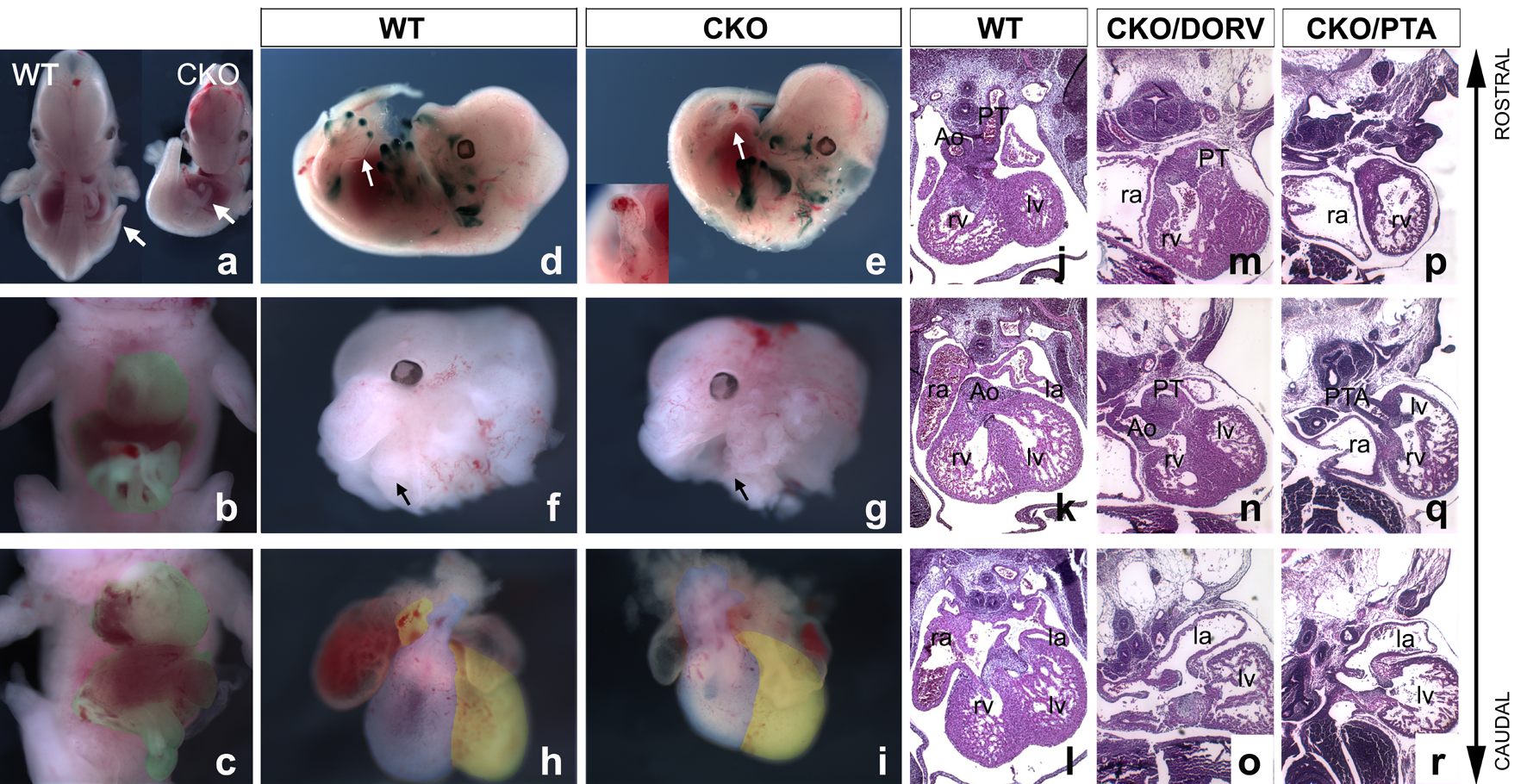

Supplement: Figure S1 — Inactivation of β-catenin in the mesenchyme resembles the phenotype of Pitx2 null embryos. (a) Frontal images of control and CKO embryos at E13.5. (b-c) Ectopic hearts and visceral organs in CKO's vs. WT with leftward displacement of ventricles (pseudo-colored in green). (d,e) β-galactosidase staining of WT or CKO embryos containing the Fgf10LacZ allele. CKO embryos display severe hind limb defects with no detectable LacZ/Fgf10 expression (inset in e). (f,g) Defective development of the mandibular and maxillary facial prominences and regression of the stomodeum. (h, i) Altered cardiac position with major cardiac outflow tract abnormalities in CKO heart (i) compared to WT (h). In WT, the pulmonary trunk (PT) rises from the right ventricle (rv) and is separated from the aorta (Ao), which rises from the left ventricle, by the aortic-pulmonary septum (rv and PT pseudo-colored in blue, lv and Ao in yellow). (j-r) Histology of control (j-l) and mutant (m-r) at E13.5 (transverse sections on comparable axial levels from rostral to caudal). Most mutants display double outlet right ventricle or DORV (m, n), i.e., both the aorta and pulmonary trunk originate from the right ventricle. A subset of mutants demonstrates a single outflow tract rising from the right ventricle, i.e., they display Pulmonary truncus arteriosus or PTA (p-r). Leftward orientation of the heart is evident in all mutants; moreover the right ventricle is largely located above the left ventricle (m-r). (4.86 MB TIF) [file pone.0001516.s001.tif]

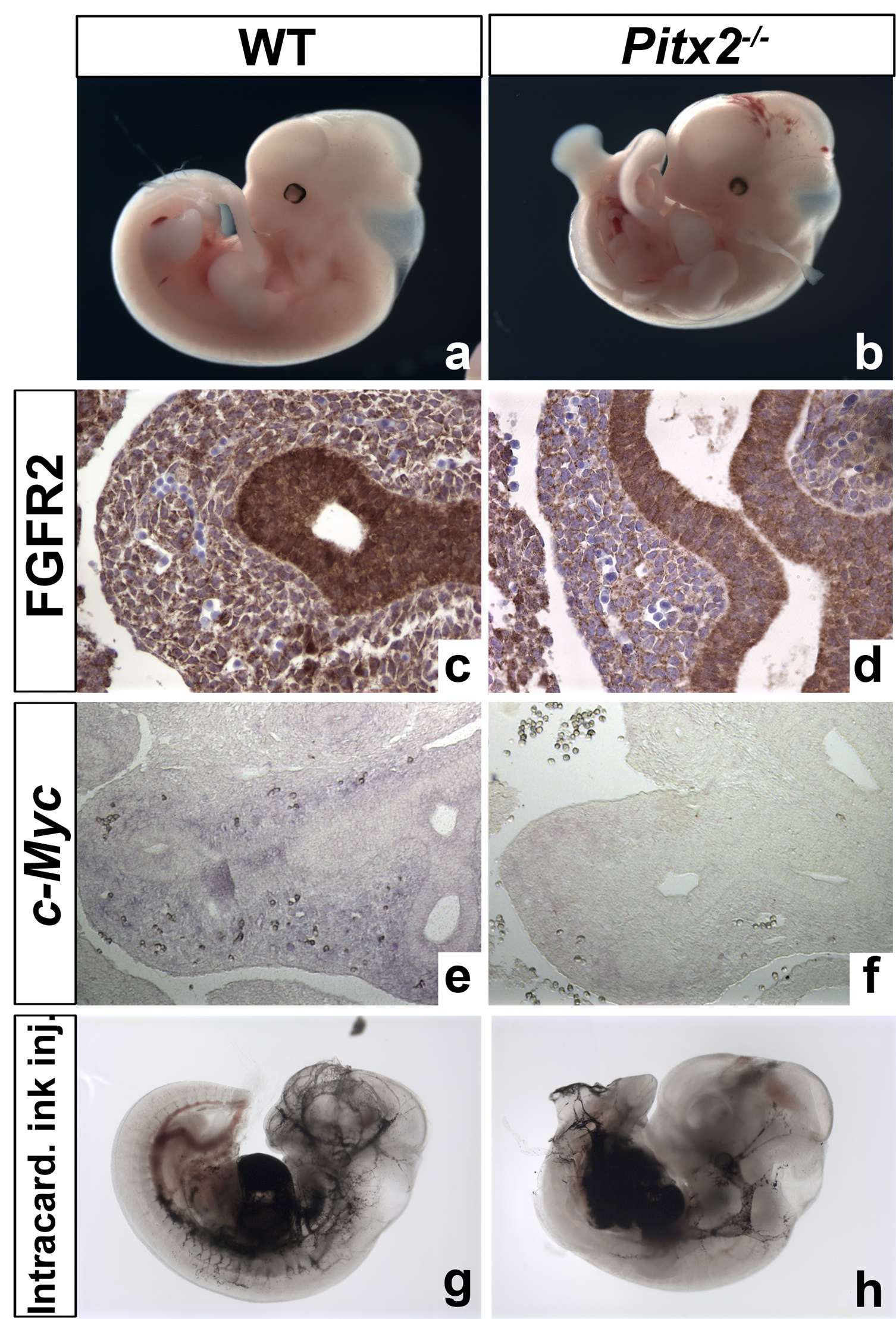

Supplement: Figure S2 — Comparative analysis of the Pitx2−/− phenotype. (a-b) Images of control and Pitx2−/− embryos at E12.5. (c-d) Immunohistochemistry. Reduced expression for FGFR2 in E12.5 Pitx2−/− lung mesenchyme and epithelium (d) compared to WT lungs (c). (e-f) Section RISH for c-Myc on E12.5 WT and Pitx2−/− lungs. Expression of c-Myc is reduced in Pitx2−/− lung mesenchyme (f) compared to WT lung mesenchyme (e). (g-h) Intracardiac India ink injection of E12.5 WT and Pitx2−/− embryos. Pitx2−/− embryos show defects in vasculogenesis and leakage of India ink from premature blood vessels is apparent (g) compared to WT embryos (h). (10.05 MB TIF) [file pone.0001516.s002.tif]
